# Photo Processing for Biomedical Hydrogels Design and Functionality: A Review

**DOI:** 10.3390/polym10010011

**Published:** 2017-12-22

**Authors:** Hongyi Yao, Jieqiong Wang, Shengli Mi

**Affiliations:** 1 Biomanufacturing Engineering Laboratory, Graduate School at Shenzhen, Tsinghua University, Shenzhen 518055, China; yaohy16@mails.tsinghua.edu.cn (H.Y.); wangjq17@mails.tsinghua.edu.cn (J.W.); 2 Open FIESTA Center, Tsinghua University, Shenzhen 518055, China

**Keywords:** hydrogels, photo crosslinking, photo degradation, photo dimerization, biomedical

## Abstract

A large number of opportunities for biomedical hydrogel design and functionality through photo-processing have stretched the limits of innovation. As both photochemical understanding and engineering technologies continue to develop, more complicated geometries and spatiotemporal manipulations can be realized through photo-exposure, producing multifunctional hydrogels with specific chemical, biological and physical characteristics for the achievement of biomedical goals. This report describes the role that light has recently played in the synthesis and functionalization of biomedical hydrogels and primarily the design of photoresponsive hydrogels via different chemical reactions (photo crosslinking and photo degradation) and conventional light curing processes (micropatterning, stereolithography and two/multiphoton techniques) as well as typical biomedical applications of the hydrogels (cell culture, differentiation and in vivo vascularization) and their promising future.

## 1. Introduction

Hydrogels are a class of hydrophilic polymer materials that can absorb large amounts of water without dissolving. The broadly tunable physical and chemical properties of hydrogels have drawn tremendous attention in the biomedical field and serve as compatible platforms or building blocks for creating biomimetic structures. As long as the materials have no vital negative biological impact, both natural and synthetic polymers can be utilized to fabricate hydrogels. With intrinsic bioactive motifs for cell-cell and cell-matrix interactions, natural materials for hydrogels fabrication (e.g., gelatin, alginate and hyaluronic acid) shows a great advantage on the maintenance and promotion of cell function. While, synthetic polymers, such as poly (ethylene glycol) (PEG), provide versatile adaptivity and controllable properties. Those features may be more beneficial in the design and modification of the matrices with more bioactive functions [1]. Conventionally, hydrogels possess a relatively poor mechanical modulus and are prone to functional failure. In addition, the lack of dynamically modifiable properties and the inability to precisely fabricate structural complexity severely hinder the development of hydrogel systems. In recent decades, investigators have tailored various properties, such as the electrical conductivity [2,3] stiffness and active cellular microenvironment [4] of bulk hydrogel substrates derived from nature [5,6,7,8] or synthesized [9,10]. 

Furthermore, high-resolution light-induced rapid prototype fabrication techniques enable scientists and engineers to create spatiotemporal, interactive hydrogels containing on-demand gradients of physical or chemical cues. Methods such as micropatterning, stereolithography (SLA) and two-photon polymerization, have been applied through various innovative strategies to perform dynamic modulation of the hydrogels formed along predefined paths and control spatial heterogeneity within the hydrogel structures. Those features impact behaviors of loaded cells [11,12,13], facilitate tissue cultivation [1,14,15] and optimize the functions of biological micro devices [13,16,17,18,19]. 

Researches that encompasses photochemistry, polymer science, light-based processing and engineering has delivered hydrogels that are remarkably low cost, highly sensitive and possess dynamic properties. In this review, we describe our vision of how such photoresponsive hydrogels can be synthesized via the chemical modification of backbones and monomers for photo curing (crosslinking) and outline the precise regulation techniques through the photodegradable strategy. We then examine in more detail the common procedures utilized in the photo fabrication of hydrogels [20]. Furthermore, we scan several typical fields that benefit from light modeling and remote photon manipulations. Finally, we discuss the limitations that need to be overcome for the photo integration strategy to fulfill its potential and broaden its commercial uses.

## 2. Rational Design of Hydrogel for Photo-Induced Construction and Modification

Generally, two kinds of photo-involved reactions are utilized for hydrogel gelation and modification. Photo-induced polymerization and degradation, both contribute to the forming and shaping capabilities of hydrogels, undergoing the exposure across the spectrum from UV to red. Here, we start with the construction of light cured hydrogel, realized by their specific end functional groups through photo initiated radical polymerization or photo click crosslinking. Those two kinds of chemical reactions consist the mainly mechanism of the gelation of the photo-induced hydrogels. The degeneration trigged by light impulse also gives the chances of shaping the hydrogel bulk, on-demand releasing drug and forming biomimetic microenvironment. It is essential that taking a short scan on this topic through several typical cases. Moreover, the photo dimerization, which combine the both advantages of crosslinking and degradation, will be discussed at the end of this section. 

### 2.1. Light Curing Hydrogels Design

We begin with a technique that turns a liquid monomer solution into a gel via light exposure, which is mainly achieved through two types of reactions, photo initiated polymerization and photo click chemistry. This technology is also called light curing. The main advantages of light curing, or UV curing, are the high polymerization rate and high conversion that can be achieved under intense irradiation since the active wavelengths are mostly focused in the range of 250–395 nm. In addition, these reactions commonly occur only in the irradiated areas and thus, relief patterns can be obtained after irradiation by using a mask and subsequently developed with solvents.

#### 2.1.1. Photo initiated Polymerization

Generated by photo irradiation, reactive species such as radicals, cations, or anions can initiate the polymerization of monomers. Once initiated, the chain reaction in liquid solution will develop very much like that in conventional thermal polymerization except with much higher kinetic rates of initiation under intense irradiation. In addition, this higher rate is one of the factors that contribute to the increasing attention on this so-called photo initiated polymerization, or photo polymerization, in studies, especially those regarding biomedical applications. Most of the research efforts for biomedical hydrogel synthesis through photo initiated polymerization have focused on highly reactive, functional monomers with potentially detailed applications.

Radical photo polymerizations are the most common and accepted type for scientists and engineers designing hydrogels composition and synthesis pathways. They can take place at mild conditions (room temperature, aqueous system, neutral pH, etc.) and allows for temporal and spatial manipulation of the reactions. Rapidly reacting with radicals, acrylates and methacryloyl substituents (MA) are the most common reactive groups utilized in radical photo polymerizations. They provide spatiotemporal control through means of a chain-growth mechanism and have a wide range of applications, from coating to scaffold forming, contact lenses, 3D cell cultures (Table 1).

With the introduction of MA, gelatin methacryloyl (GelMA), which is also referred to as gelatin methacrylate, methacrylated gelatin, methacrylamide gelatin, or gelatin methacrylamide, gains a superior photo crosslinking ability due to the radical photo polymerization of the methacryloyl groups with the assistance of a photo initiator under UV light exposure. In addition, since Arg-Gly-Asp (RGD) motifs and matrix metalloproteinase (MMP) are nearly independent of the MA groups [21,22], GelMA maintains good cell adhesion and cell-mediated enzymatic degradation, thereby mediating the hydrogel cellular properties, such as migration, proliferation and differentiation. Those advantages give GelMA great promise as bioprinting inks [23,24] and extracellular cell matrix (ECM) mimics [25]. Typically, GelMA hydrogel networks have good stability, with macromere DoF (degree of functionalization) values of 20%–80%, high stiffness and durability and an enhanced porous distribution. Upon varying the GelMA macromere concentration from 5% to 20%, the compressive modulus sharply increases from 5 to 180 kPa [5]. 

Similar modifications can also be obtained with hyaluronic acid and other polysaccharides. There are several active groups on the hyaluronic acid (HA) backbone, such as carbonyls and acids, which can be modified to form methacrylated HA. As with GelMA, by varying the macromere weight, number of reactive groups and concentration of macromeres, the mechanical properties of HA hydrogels can be tuned across several orders of magnitude. Furthermore, interpenetrating networks (IPN) can be obtained through the gelation of collagen (or fibrin) and the photo initiated radical polymerization of methacrylated hyaluronic acid (MAHA), which spatially manipulates the mechanical properties of the networks [26]. Christine E. Schmidt and her group spatially manipulated the photo polymerization of GMHA (HA methacrylated with glycidyl methacrylate) within protein-based matrices, which provided simultaneous topographical and biochemical guidance to cells in a single step [27]. The multiphoton polymerization technology used in that project will be discussed further in the following sections. 

**Figure 1:**
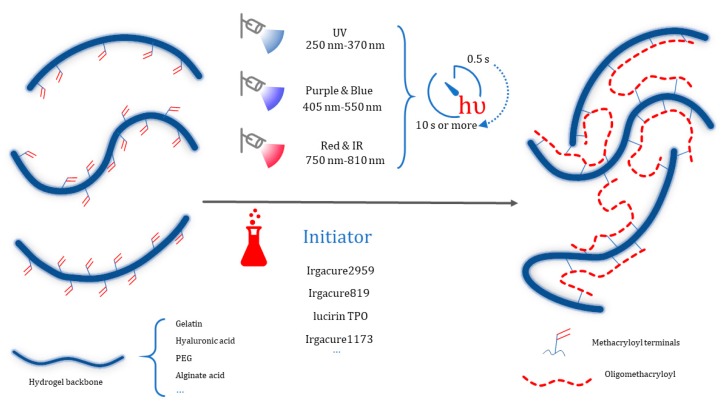
Upon generation of a free radical (by different types light exposure that depend on the type of photo initiator), the methacryloyl terminals on hydrogel backbone chains, such those of as GelMA, hyaluronic acid MA and PEGDA, polymerize to generate a more connected network through the formation of short oligomethacryloyl chains.

Poly (ethylene glycol) (PEG), which is hydrophilic, relative inert and broadly adaptive, has become an ideal “blank slate” material for tailoring and constructing hydrogels with specific mechanical and biochemical properties. With respect to photo initiated radical polymerization, dimethacrylated PEG (PEGDA) and its multi-armed derivatives form hydrogel networks through a chain-growth mechanism. Polymerization is initiated by radicals generated from the photo cleavage of initiator molecules, which propagate through the unsaturated vinyl bonds on the PEGDA macromolecular monomers. Chain polymerization occurs by continuing this process (Figure 1).

Photo initiation has accelerated the network quality of GelMA, MAHA, PEGDA, etc., which decreases the possibility of cell damage, nearly eliminates the time-consuming drawbacks of thermal radical polymerization and provides a novel ability to spatially manipulate the generation of hydrogel networks. However, the chain-growth mechanism limits the sensitivity of the hydrogel response to light exposure. In addition, the addition of proper initiators increases the complexity of the procedures for preparing the material and the possibility for potential hindrances of cell activity. Fortunately, this light-accelerated polymerization can provide stable, biocompatible and state of the art hydrogel backbones. In addition, photo click chemistry provides a complementary method for further improving the light-curing approach. 

#### 2.1.2. Photoclick Chemistry 

Photoclick chemistry offers a rapid, highly selective, versatile and photoresponsive methodology for combining multiple components and functionalizing hybrid hydrogel systems. The applications of bio-orthogonal click reactions have increased in the last decade. In particular, since researchers have provided a better understanding of the photo-induced mechanism and photo initiator design, several adaptive click reactions, such as thiol-ene reactions (including Michael additions) [28], strain-promoted azide-alkyne cycloadditions (SPAACs), the Diels-Alder (DA) reaction [28] and its derivatives [29], hydrazone reaction [30], etc., have provided an increasingly broad and versatile range of synthetic strategies for hydrogel biomaterials through light regulation directly or indirectly. 

Thiol-ene and thiol-yne click chemistry, including thiol-based Michael addition, has become the dominant class of photo click chemistry since Hoyle and Bowman pioneered this field in 2010 [31]. They showed the great adaptivity of the spatial and temporal regulation on the photo click reactions for polymer synthesis. Light-regulated thiol-ene radical reactions improve the overall simplicity and compatibility and maintain the classic advantages of photo initiated click reactions, which can be achieved at specific locations and times with high precision through the connection of step-growth and chain-growth processes. This hybrid polymerization significantly optimizes the kinetics, reduces extra shrinkage and stress and in particular, increases the insensitivity to oxygen inhibition. Han Shih, Chien-Chi Lin and co-workers developed a novel multilayer hydrogel construction process with the retention and prolonged release of eosin-Y, a visible-light initiator [32]. Through this special catalytic process, researchers simplified the thiol-ene and PEGDA interfacial photo polymerization reactions developed before and accelerated the reaction kinetics by the step-growth click gelation mechanism. Different from the chain-growth mechanism, step-growth gelation occurs when two or more multifunctional monomers, such as PEGDA and Gel-norbornene, mutually react in either an imbalanced ratio or a stoichiometric balance [33]. 

Basically, two types of click reactions have emerged in the field of thiol-ene photo curing applications (Table 2); thiol-(meth)acrylate free-radical addition to electron-rich/electron-poor carbon–carbon double bonds [31] and thiol-norbornene photo polymerization combine the benefits of radical-mediated polymerization and the bio-orthogonal click reaction [22,23]. It is worth noting that those click reactions described above can occur in a proper aqueous environment within several minutes without the involvement of light, yielding homogeneous crosslinked hydrogel networks. However, once those mutually reactive monomers are mixed together, “click” crosslinking occurs. The relatively long response time and uncontrollable features do not meet the requirements of many engineering situations, such as bioprinting and drug release, which urgently require spatiotemporal manipulation or fabrication. In the presence of radicals generated from the photoexcitation of initiators, multifunctional monomers are more reactive and efficient, with the reaction generally occurring within several seconds to form crosslinks under mild conditions, which may not suitable for photo-involved click reactions. 

As we mentioned above, gelatin can be modified with methacryloyl (GelMA) and homopolymerized via chain-growth photo initiated polymerization in the presence of an initiator. Furthermore, GelMA can also be more intensively crosslinked with multifunctional thiols (e.g., dithiothreitol (DTT), 4arms-PEG-HS) through thiol-acrylate or Michael addition. In addition, gelatin can also be thiolated and crosslinked with low-molecular-weight linkers, such as PEG-diacrylate (PEGDA), to form hydrogel networks via a thiol-acylate click reaction. Through this method, Weiyuan John Kao and his group members developed a hybrid biomatrix based on the thiol-acrylate reaction of PEG diacrylate (PEGDA) and cysteine/PEG-modified gelatin (gel-PEG-Cys) [45]. Weitao Jia, Ali Khademhosseini and their group utilized the thiol-acylate photo click addition for the post-modification of alginate-calcium covalent hydrogel structures printed through a coaxial nozzle device. This sequential photo manipulation strengthened the entire elastic modulus of the network and prolonged the cell culture period to more than 20 days [23].

The thiol-norbornene photo click reaction occurs through a unique polymerization mechanism that combines the advantages of radical-mediated step growth polymerization and a bio-orthogonal click reaction. This hybrid reaction accelerates the polymerization rate and minimizes the chemical toxicity that may induce potential biological damage. Moreover, with additional regulation of the spatial and temporal light exposure, thiol-norbornene hydrogels can be modified with more physical (e.g., elastic modulus) or biochemical (e.g., cell adhesion peptides) properties. Chien-Chi Lin and his group developed the photo initiated crosslinking and hydrolytic degradation of a thiol-norbornene PEG hydrogel system [47] and expanded this idea across several biomedical fields, such as visible-light initiation [32], drug delivery [48] and 3D cell culture [46]. They found that compared to hydrogels prepared by Michael addition (e.g., thiol-(meth)acylate hydrogels), the thiol-norbornene hydrogels formed with faster gel points and a higher crosslinking density. In addition, the presence of ester bonds within the 4arms-PEG-norbornene macromeres accelerated the hydrolytic degradation of the hydrogel network [47]. Then, by combining with the tetrazine-norbornene inverse electron-demand Diels-Alder reaction, Chien-Chi Lin and his group members performed thiol-norbornene photo click reactions as a sequential modification method for realizing cell-instructive hydrogels and specific protein pattern platforms for further cell differentiation research [46]. Recently, these researchers modified the thiol groups with cyclodextrin (CD), yielding a photoinitiation (365 nm)-generated and photo-regulated (430 nm) releasing hydrogel network [48]. With the rise of thiol-norbornene photo click reactions and other photo-free click chemistry, the classic thiol-vinyl or thiol-based Michael-type click reactions, which can be initiated or accelerated by light exposure, are utilized as complementary or post-gelation techniques to impart hydrogel networks with more spatially or temporally focused biochemical functions.

**Figure 2:**
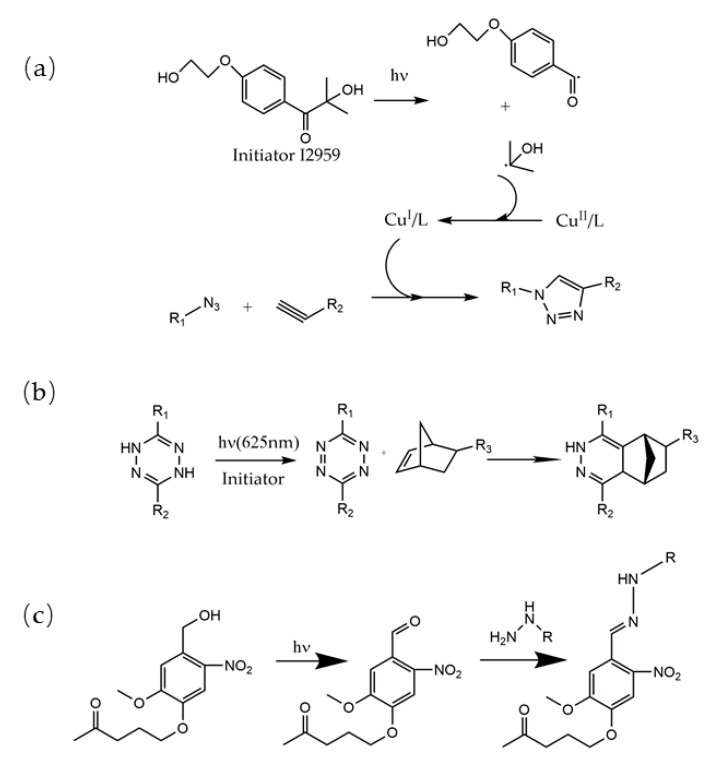
Several indirect photo regulation strategies combining click reactions and photochemistry, such as (**a**) redox reduction of Cu(II) to Cu(I) via photo initiator radicals [43], (**b**) light oxidation [50] and (**c**) photo cleavage via hydrazone adduction [30].

Furthermore, the inclusion of light in gelation can trigger the next polymerization steps, which may also be initiated by stimuli other than photons, such as heat, magnetics and even strong bases. (Figure 2) Malar A. Anagram and his group synthesized a unique PEG hydrogel using photo cleavage-driven step-growth polymerization. With UV exposure, the hydroxyl groups on the nitrobenzyl terminals were cleaved to form aldehyde groups, which rapidly reacted with hydrazine-functionalized PEG in mild aqueous conditions [30]. Benjamin W. Muir, Rodney T. Chen and their co-workers reported that photo-generated radicals and UV absorption by a copper chelating ligand resulted in the photochemical redox reduction of Cu(II) to Cu(I), which catalyzed the alkyne-azide click reaction to form an azide-functionalized plasma polymer hydrogel film. Through photolithographic patterning, the PEG hydrogel film showed spatial resistance to the adhesion of L929 mouse fibroblast cells in a parallel line distribution, showing a novel process of hydrogel surface modification via alkyne-azide and photocatalyst click reactions [43]. Moreover, Vinh X. Truong, John S. Forsythe and their group reported the utilization of red-catalyzed oxidation of dihydrogen tetrazine for the activation of tetrazine-norbornene inverse electron-demand Diels−Alder conjugation, which occurred rapidly under physiological conditions to form biocompatible hydrogels [50].

Although radical photo initiated polymerization and photo click conjugation provide possibilities for the spatial and temporal control of the photo curing process of hydrogels, the potential toxicity, negative limitations and undesirable byproducts generated from the reactions of the catalysts, initiators and auxiliary ingredients still effect the final physiochemical and biochemical performance of the cured hydrogels. Meanwhile, the occurrence of many bio-orthogonal reactions, such as Diels-Alder reactions and strain-promoted azide-alkyne cycloadditions (SPAAC), which can rapidly occur in mild, aqueous ambient conditions, are independent of the presence of light. Those features challenge the fabrication of heterogeneity and micro-architected biomedical hydrogels. 

However, there should always be a solution to the problem. Photo removable protecting groups (also known as caging groups), such as *o*-nitrobenzyl derivatives and 6-bromo-7-hydroxy coumarin (Bhc), may can be used to mask the specific functionalities present in bioactive agents (yielding inactive caged molecules) and these protecting groups can be removed selectively upon irradiation to unmask the active groups for crosslinking. Through this method, important connection moieties, such as those utilized in the photo-independent click reactions mentioned above, supramolecular systems and enzymatical conjugation can be achieved within the photo cured hydrogels.

### 2.2. Photodegradable Hydrogels Design

Photodegradable properties provide hydrogels with more alternatives for post-gelation strategies, such as temporal changes, the creation of arbitrarily shaped features and target-dependent or on-demand functionality release [51]. Generally, *o*-nitrobenzyl (*o*-NB) linkers are the dominant basic unit of photodegradable hydrogel monomers since they are easily incorporated without requiring the use of small molecule catalysts or other toxic compounds. 

In recent years, *o*-nitrobenzyl (*o*-NB) alcohol derivatives have gained tremendous attention among other studied photo labile groups in the synthesis of hydrogels by photo degradation or photo cleavage. Under UV light irradiation, an *o*-nitrobenzyl alcohol derivative photo isomerizes to the corresponding *o*-nitrosobenzaldehyde, immediately releasing free carboxylic acid. Andrea M. Kasko and his group synthesized *o*-nitrobenzyl (*o*-NB) linkers on PEG4k-DA macromonomers in the utilization of maskless photolithography as a facile process for the direct, noncontact gradient patterning with custom graphics. Under a gradient of exposure intensity, the hydrogel showed a spatially resolved modulus, resulting in different guidance of the migration behaviors of human mesenchymal stem cells (hMSCs) [52]. Eben Alsberg and Vincent M. Rotello reported a similar study that added *o*-NB units to a PEG-based hydrogel system. Compared with the kinetics of hydrolysis, the RNA release rate from the photodegradable hydrogel was accelerated via UV excitation, resulting in a more efficient osteogenic differentiation of hMSCs [53]. In addition to the photo labile group modification, photoresponsive supramolecular amphiphiles based on host-guest molecular recognition motifs also have potential in photo-based hydrogel biomedical applications, which have been reviewed in detail before [54].

Molly S. Shochet and her group synthesized bromo-hydroxycoumarin-protected (Bhc) thiols and conjugated the compounds to agarose hydrogels. Upon two-photon irradiation (without initiator), the Bhc groups undergo photo cleavage to liberate thiols, which then react to immobilize bioactive maleimides, achieving spatial orientation within the hydrogels [55]. Recently, the researchers improved the efficiency of two-photon patterning in three-dimensional HA hydrogels by using a 3-methyl-6-bromo-7-hydroxy coumarin (mBhc) photo labile group, which decreased the irradiation power and enhanced the bioactivity [56]. Moreover, for temporal control, photo labile groups can be used to mask the bioactivity of proteins and peptides until the cages are cleaved by photo irradiation, which promotes the realization of fully biomimetic hydrogels [57,58].

### 2.3. Photodimerization Hydrogel Design

**Figure 3:**
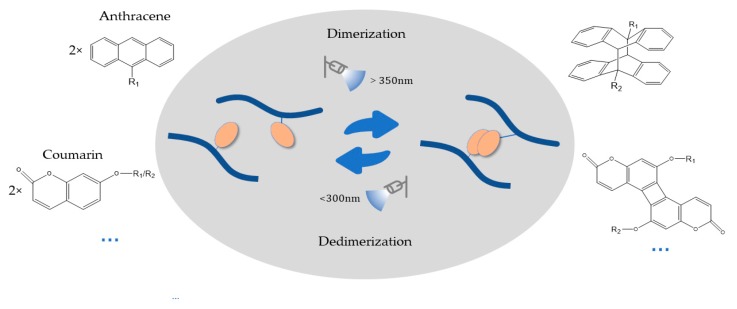
Mechanism of photo dimerization. The monomers (e.g., anthracene, coumarin) dimerize under UV exposure at wavelengths larger than 350 nm and divide under irradiation at wavelengths below 300 nm.

In special cases, photo dimerization can be utilized as a crosslinking mechanism under UV light exposure with wavelengths above 300 and the same functional groups can be degraded under irradiation by a shorter wavelength of light (Figure 3). This catalyst-free reversibility provides a broader range of spatiotemporal manipulations and minimizes the potential cell damage of biomedical hydrogels. 

Anthracene, coumarin, thymine, cinnamylidene acetate and nitrocinnamate are common dimerization groups investigated in catalyst-free photo crosslinking. With their superior characteristics, such as photo reversibility and rapid, catalyst-free click reaction, these groups have drawn the interest of many kinds of fields, such as drug release, tunable mechanical responses, initiated self-assembly by wavelength changes and self-healing material synthesis. 

Anthracene is one kind of well-understood photocycloaddition molecule whose detailed photo properties have been researched for many decades. The [4π + 4π] photocycloaddition of anthracenes forms a bio-orthogonal benzene molecule [59,60,61,62] and occurs under irradiation at wavelengths larger than 300 nm. Since anthracene is highly hydrophobic, improving its solubility in water or choosing a suitable hydrophilic backbone is essential for the function of hydrogel-anthracene photoresponsive systems. PEG chains, an ideal material with various end groups and different types of configurations, can act as efficient connectors or backbones to incorporate anthracene molecules into hydrogel systems and increase their photo reactivity. Laura A. Wells and her group grafted a generic PEG-anthracene crosslinker onto a hyaluronic acid backbone and treated it with 365 nm light of various intensities (12,000, 18,000, or 24,000 mJ·cm^−2^). The results showed that light exposure could turn off the release of myoglobin from the bulk hydrogel [59]. In addition, several groups have placed electron-rich substituent groups, such as triazole and benzyl triazole, at the 9-position of anthracene, resulting in a redshift in the absorption of anthracene to the wavelengths of visible light (400–500 nm) and an improvement in the simplicity of the procedure for 3D cell culture studies [63,64]. Furthermore, the catalyst-free photo crosslinking power is illustrated by the tunable control of the mechanical properties of thermoplastic elastomer hydrogel networks, which is determined by the ratio of ABA triblock copolymers to AB diblock copolymers. Nabila A. Huq and her group members recently added terminal anthracene units to a ω-anthracenylpolystyrene-β-poly (ethylene oxide) diblock copolymer precursor (SO-anth) to produce a certain controllable degree of SOS triblock polymer. Under various UV exposure times ranging from 2 to 20 min and a constant irradiation intensity of nearly 30 Mw/cm^2^, the ABA/AB hydrogel system showed a tunable ratio (from 11.7 to 45 mol %) and different modular and mechanical responses [65]. 

In cooperation with self-assembled dipeptides, a light-triggered enhancement of nanostructured hydrogels could be achieved by photo dimerization. Se Hye Kim and her group functionalized a lysine dipeptide with 7-(diethylamino)-3-coumarin carboxylic acid (7-DAC) to form a self-assembled hydrogel comprised of uniform micrometer-length nanofibers. Under irradiation at 365 nm, the nanofiber network, dominated by π stacking, could be crosslinked and stabilized by the dimerization of the coumarin groups [66]. 

In addition to photo-induced crosslinking, photo dimerization also has great potential for application in photodegradable and self-healing functional material design with shorter wavelength light (254 nm) [62,67,68]. For instance, Pablo Froimowicz and his colleagues designed a superior method for synthesizing a healable material with the reversibility of photo dimerization. When the specific hydrogel film was damaged on the surface, the researchers put it into a solution of the monomers and irradiated the material at 254 nm, which induced the degradation of the crosslinked hydrogel into hyperbranched macromonomers, polyglycerol (PG-An), modified by anthracene end groups. Finally, a freshly regenerated bulk hydrogel film was achieved by the photo crosslinking of those dendritic nanometer-sized building blocks at 365 nm to complete the healing process [62]. 

## 3. Processing

In the following sections, we introduce different microscale techniques for the fabrication of hydrogel constructs. These include micropatterning, stereolithography and two-photon polymerization, which can be used to control the geometrical features as well as the distribution of cells within the fabricated hydrogel constructs (Table 3).

### 3.1. Micropatterning

#### 3.1.1. Working Principles and Traits of Micropattern

**Figure 4:**
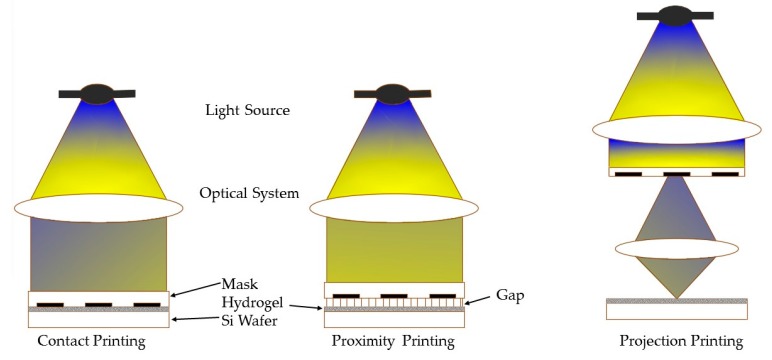
Schematic of the micropatterning technique. The type of the micropattern is divided into contact printing, proximity printing and projection printing due to the different exposure modes.

Micropatterning is a technique with easy-to-use equipment and operating steps that was originally developed for the fabrication of constructs with micron-sized features. Fabricating hydrogel constructs using the micropatterning technique requires a light source (e.g., UV, blue or red light), a photomask and a photocrosslinkable hydrogel mixed with single or multi photo initiators [69]. The scheme of the micropatterning technique shows in Figure 4. A photomask is a two-dimensional sheet that generally includes both transparent and opaque areas and of course, only the transparent area of the mask allow light passes through [39,70,71,72,73]. The light acts as the necessary stimulus to trigger the photo-induced reaction and crosslink the monomers or prepolymers. The portions of the prepolymer hidden by the opaque regions of the mask remain in the liquid form and can be removed by washing with a developing agent. The generated solid polymer patterns are planar and the process can be repeated to form 3D features with different masks in a series of ordered steps [74]. The crosslinking reaction can occur within seconds, or small fractions of a second. It is a rapid process and the reaction time depends on the relative concentrations of the monomers, prepolymers and photo initiator as well as the intensity and duration of the light exposure [54]. 

#### 3.1.2. Hydrogel Feasibility and Limitations for Micropattern

There have been many kinds of hydrogels used with the micropatterning technique and these hydrogels generally contain acrylate groups in their backbones [89]. Photocrosslinkable hydrogels such as PEGDA [75], PEGDM (PEG-dimethacrylates), GelMA [90], methacrylated HA and MeTro [76] can be used for micropatterning. PEGDA hydrogels have demonstrated the ability to maintain the viability of the encapsulated cells and the integrity of the constructs and reliable feature sizes can reach below 100 µm [70,91]. Photodegradable hydrogels can also be used for micropatterning. For example, by conjugating photodegradable *o*-NB (orthonitrobenzyl) groups to PEG, Wong et al. formed a photodegradable hydrogel [92]. 

Using micropatterning to fabricate microscale constructs is a simple and versatile approach that can precisely control the spatial microenvironment of cells. With a high solution greyscale distribution [93], or customized linear movement [94] or rotating [95], photomask can be utilized to create single or dual gradient substrate [96]. Those substrate with gradually changing physical properties, immobilized proteins and soluble cues, mimic the microenvironment in vivo and may guide the cell directional migration in vitro. It can also produce 3D cell-laden constructs that form various cells through continuous photopatterning of a hydrogel containing different cell types. However, the properties of the prepolymer, such as the solvent that the prepolymer is dissolved in, can be cytotoxic in cell encapsulation. The control of an appropriate UV exposure time is difficult and it is reported that longer exposure times can affect the viability and activity of cells and reduce the fidelity of the desired pattern [89]. The resolution of the fabricated constructs depends on the quality of the photomask and the illumination system that is used and it can also be limited by the aspect ratio. In addition, it is challenging to develop the patterns with the masks when cell distribution is required [69]. 

The energy required in the fabrication process is usually delivered by UV light. It has been demonstrated that other wavelengths can also be used [97,98]. For example, by adding eosin Y and triethanolamine, PEG-based hydrogels could be crosslinked under visible light [97] and the resulting viability of the encapsulated human mesenchymal stem cells increased by 10% [90,99]. 

### 3.2. Stereolithography (SLA)

Stereolithography (SLA) was originally described by Charles W. Hull in his 1986 patent (US Patent 4,575,330) and it is an additive-based fabrication technique for sequentially joining materials or energy to form a construct from 3D model data. SLA is also an RP (rapid prototyping)-based method and the fabrication process usually spans only a few hours, whereas it could take several hours or even several days with other methods [89]. SLA systems are automated, and the basic process involves four steps. First, the construct is previously designed using a CAD program [100]; second, the CAD files are converted to STL format, which describes the actual surfaces through raw unstructured triangulated surfaces; then, the STL files are converted to sliced models; finally, the construct is fabricated using the machine layer-by-layer [101]. The thickness of the thin cross-sectional layers can be varied from 0.01 to 0.7 mm depending on the SLA technique [100]. 

#### 3.2.1. Working Principles and Traits of SLA

**Figure 5:**
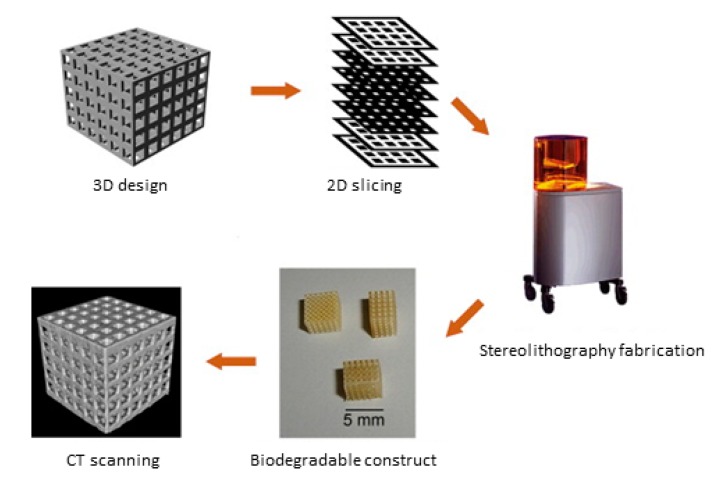
Overview of the processes that the constructs are fabricated using SLA. Reproduced with permission from Reference [79].

An SLA device consists of four parts, thus, a reservoir filled with a liquid photosensitive material, a laser source (commonly UV light with a wavelength of 325 nm), a fabrication platform moved in the vertical plane and a system that controls the XY movement of the light beam. Scanning the surface of the photosensitive material orderly, the local exposure region of the liquid level produces 2D patterns of polymerized material via single photon absorption mechanism [101]. The hydrogel in the focal plane are crosslinked in point format generated by the sliced model [69] and the platform moves incrementally in the Z direction after a predefined period, concerning the kinetic of hydrogel crosslinking. This process is repeated until the construct is complete [102]. Post-treatment is essential in most cases after fabrication, including washing off the excess material and further curing with UV light [101] (Figure 5).

**Figure 6:**
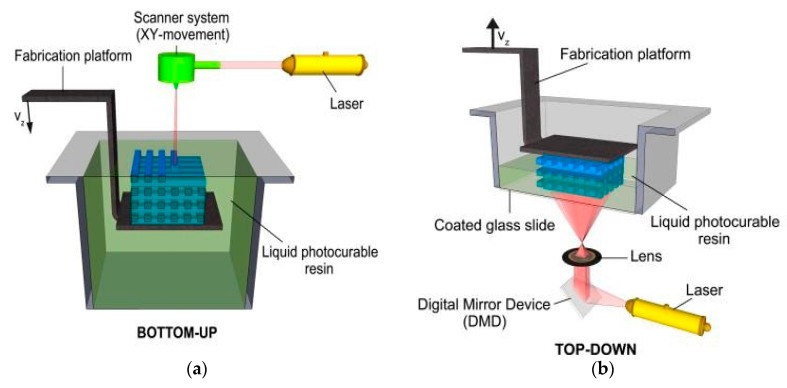
Schematic of two kinds of SLA approaches. (**a**) Bottom-up setup. In the top-down setup (**b**), every newly fabricated layer is underneath the previous layers and the polymerization of the light-sensitive material is performed by irradiation from underneath. Reproduced with permission from Reference [101].

The SLA device may have two kinds of configurations. One is called the bottom-up setup and the other is the top-down setup. Figure 6a shows a scheme of the bottom-up setup. In the bottom-up version, constructs are fabricated from a support beneath the resin liquid level and the subsequent layers are cured by irradiation from above the previous layers. The bottom-up setup is widely used so far [82,84,103,104,105,106]. While, the breakup of the equilibrium of the liquid level caused by every single movement of the support along the Z axis, costs extra time for recovery to state, sacrificing the efficiency of the whole procedure. Thus, the top-down setup, which is shown in Figure 6b, shows great potential [79,107]. In the top-down version of the SLA, the fabrication platform moves from top to bottom, which is the opposite direction of the other approach and every newly fabricated layer is underneath the previous layers. The photo-induced polymerization of the hydrogel material is carried out by irradiation from underneath. It is noted that the irradiated part of the fabricated construct is prohibited being exposed to the oxygen from the surrounding, inhibiting unnecessary crosslinking. Since there is no disturbed from the movement of the support, the surface of the fabricated construct is always very smooth and does not require modification [79]. 

#### 3.2.2. Hydrogel Feasibility and Limitations for SLA

SLA is one of the typical laser-based RP strategies [79]. While, This technique is only applicable to photocrosslinkable hydrogels because of the crosslinking of the hydrogel can only occur in the focal plane and the rest of the hydrogel will always remain aqueous and uncrosslinked [69]. In 2003, Cooke et al. first reported the fabrication of biodegradable scaffolds by SLA. In this case, the structures were made using a biodegradable resin mixed with diethyl fumarate, poly(propylenefumarate) and bisacylphosphine oxide, acting as photo initiators. This approach was also adopted to fabricate photo labile hydrogels for possible soft tissue engineering applications, along the attempt to apply SLA on hard tissue engineering applications [103,107,108].

Unlike mask-based micropatterning, which is limited by its high cost, time-consuming nature and lack of automation in the fabrication of larger constructs, SLA can get over these challenges through its unique procedure properties, thus, maskless, high throughput and substrate-specific [79,109]. In general, SLA is a valuable method for fabricating complex 3D architectures to guide cellular alignment and behavior within a fabricated construct [5]. However, SLA is limited by its requirement for appropriate photocrosslinkable materials and suitable UV exposure times. The largest drawback is its limited resolution due to the shrinkage of the scaffolds that occurs post-processing [82]. In addition, since the entire area should be scanned for each focal plane, SLA is a slow process that is harmful to cell encapsulation [69]. In general, the use of SLA in tissue engineering applications is restricted by its drawbacks [100]. 

Microstereolithography, called µ-SLA, was introduced to counter the finite resolution of SLA. The working principle of µ-SLA is the same as that of conventional SLA. For example, Lee et al. [81,82] used an alginate hydrogel and an acrylated trimethylene carbonate/trimethylolpropane framework to fabricate a hybrid scaffold for the encapsulation of chondrocyte and demonstrated that the encapsulated cells maintained their original phenotypic expression in the construct. The scaffold remained mechanically stable for 4 weeks after transplantation in test mice. 

### 3.3. Two-Photon Polymerizations (TPP)

#### 3.3.1. Working Principles and Traits of TPP

**Figure 7:**
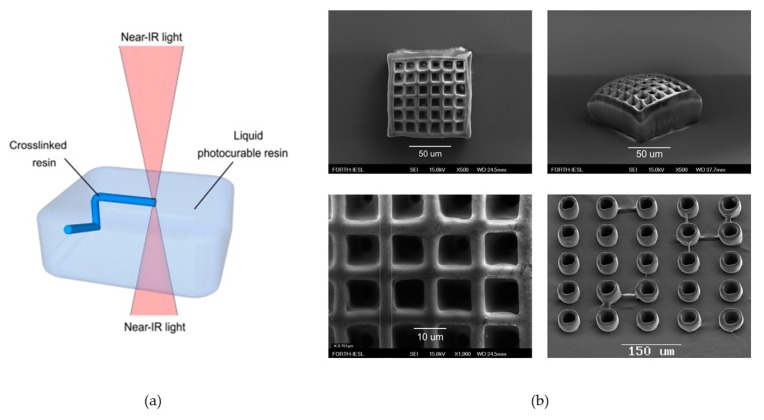
(**a**) Working principle of the two-photon photo polymerization technique. In the focal spot of the near-infrared laser beam, the photo initiator is excited and the photosensitive polymer is crosslinked; (**b**) 3D structure fabricated using two-photon polymerization. Reproduced with permission from Reference [101,110].

Two-photon polymerization (TPP) is an emerging, mostly advanced, laser-based technique for the fabrication of 3D hydrogel constructs. It has two laser pulses with long wavelengths and each of the two laser pulses acts as a single pulse with a short wavelength to excite the photo initiator and crosslink the hydrogel at the crossing point (Figure 7). In this fabrication process by the TPP technique, a near-IR (near-infrared) laser beam is used to trigger a chemical reaction, thus causing polymerization of the photosensitive material and the polymerization only occurs in the focal spot, with other areas completely unaffected. The resolution of this approach is below the diffraction limit of the applied light because of its characteristic of potential solidification and by moving the laser focus, a 3D object can be fabricated [101,110].

#### 3.3.2. Hydrogel Feasibility and Limitations for TPP

To achieve a higher resolution in the developed scaffold, TPP can offer a suitable alternative to SLA. Since this technique was only recently introduced, there are currently only a few reports that use TPP with hydrogels. Table 4 summarizes several improvement details of TPP processing achieved recently. Schade et al. fabricated hydrogel-like scaffolds with methacrylated polyurethane and PEG-DA as starting materials and demonstrated that the scaffolds had defined 3D structures. The TPP system was also used to create patterns of various biomolecules in an agarose-based hydrogel that was modified with coumarin-caged thiols [111]. Christian Peters and his group developed a superparamagnetic hydrogel microrobots, composed of magnetite (Fe_3_O_4_) nanoparticles, high-stealth PEG-DA and pentaerythritol triacrylate (PE-TA). With the assistance of TPP, they gained helical swimming hydrogel fibers, being corkscrew propelled within a weak, rotating magnetic fields. When those micro devices attached on a model cell line (3T3 fibroblast cells), they released the contained drugs and hydrolysis in aqueous [112]. 

In contrast to SLA systems, which possess a focal plane, TPP systems have a focal spot [69]. The accuracy of TPP is higher than that of SLA and the TPP technique offers higher precision and resolution in comparison with its photomask-based counterparts. However, the geometrical nature of gelation volume of the two-photon excited radicals from the P2CK, the common IR two-photon initiator, limits the vertical approximation much larger than that along the horizontal axis, resulting in that the voxel (the volume pixel), is ellipsoidal rather than spherical [112,113]. The operating systems of TPP run slowly. The long time exposure and cytotoxic component from the initiators makes the cells encapsulated in hydrogels damaged or even worse [114].

## 4. Biomedical Applications

There are tremendous studies showing that with proper photo properties, such as intensity, exposure time, the engineered hydrogels showed high fidelity and high initial cell viability. Here we focus on the role of the light played in the process of hydrogel construction and consequently, the spatially and temporal advantages on biomedical applications than none photo-involved situations. 

### 4.1. Three-Dimensional Cell Culture and Cell Behavior Research

**Figure 8:**
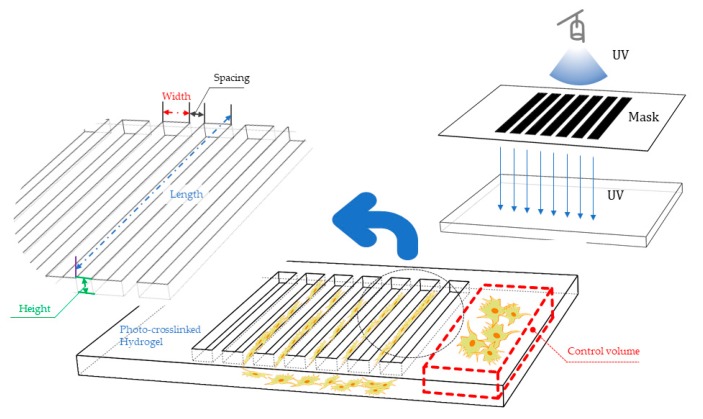
Layout of the grid-shaped micropattern and the process of lithography on the photocrosslinkable hydrogel.

**Figure 9:**
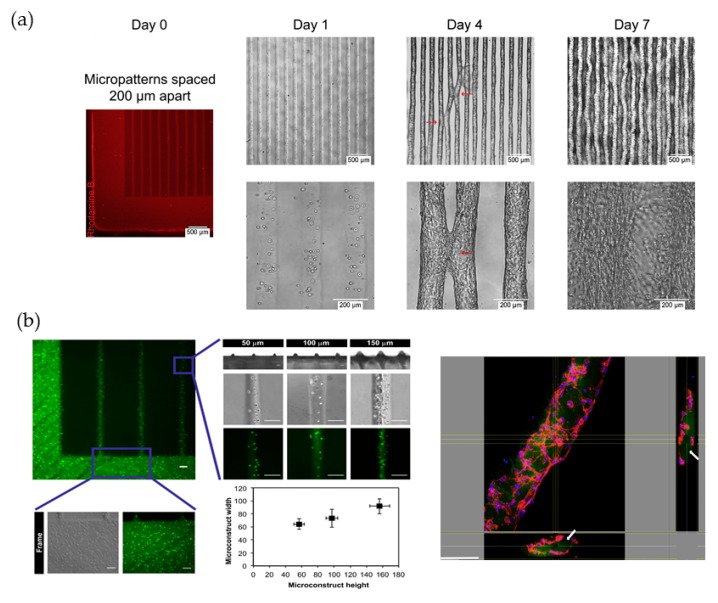
(**a**) Self-assembly of multiple micro-constructs to form a macroscale, aligned 3D tissue construct. Reproduced with permission from Reference [11]; (**b**) Micropatterned cell-laden GelMA constructs comprised of micro-constructs with variable heights (50 mm, 100 mm and 150 mm) and representative 3D confocal images of the inner layer of the cord formed within the hydrogels after 5 days of culture. Scare bars represents 100 μm in (**b**). Reproduced with permission from Reference [12].

Generally, micropatterning of photocrosslinkable hydrogels provides flexible platforms that are convenient and efficient for observing dynamic cell behaviors in 3D cell culture and for exploring cell responses of spreading, alignment, proliferation and differentiation to spatiotemporal photo-induces. In the photo initiated polymerization of biocompatible natural derivative hydrogels, such as GelMA and HAMA, grid-type geometries are frequently adopted in the design of the patterned masks. Since it is simple and has a tunable width, length, space and height layout (Figure 8), grid lithography has significantly promoted the studies of cell behaviors in various fields [2,11,12,43]. Hug Aubin, Ali Khademhosseini and their group reported a simple and direct method for controlling the alignment and elongation of fibroblasts (3T3), myoblasts (C2C12), endothelial cells (HUVEC) and cardiac stem cells (CSP) encapsulated in 3D GelMA micropatterned by external photon stimuli. First, after the essential cell encapsulation and cultivation confirmation in the photo cured GelMA, the researchers varied the width of the rectangular patterned microgeometries from 50 to 200 μm while maintaining the same height and length for all patterned hydrogels. The results demonstrated an increase in the cell alignment and nuclear elongation. More interestingly, the researchers found that the width of the cell-laden micro-constructs tended to increase with increasing culture time, which indicated that by reducing the spacing between the rectangular microgeometries and utilizing a sufficient culture time, customized macro-scale, cell-laden hydrogels with photo-manipulated cellular alignments and elongation in all three dimensions could be created, which resulted from the contact between and merging of each individual parallel line [11]. Similar to the discovery of the effects of the width, two years later, the researchers explored the impacts of various heights (50, 100 and 150 μm) of the grid-type photo patterned GelMA. An increase in the cell alignment and elongation was again observed with increasing height. More notably, the results showed that the 100-μm-high grid micro-constructs provided an optimal microenvironment for the formation of stable circular cords, which are the crucial feature of vascularized network formation [12] (Figure 9). Moreover, varying the UV exposure time [43] and optimizing other pattern types (e.g., hexagon, circle [2]) endowed the hydrogels with extra physiochemical functions, such as electrical conductivity with carbon nanotubes (CNTs) and maintenance of the excellent cell viability.

In addition to micropatterning, two-photo polymerization of three-dimensional hydrogels is also a promising approach for studies of cell mechanical behavior. Hydrogel scaffolds with micrometer and sub-micrometer resolution are ideal platforms for the thorough investigation of the molecular mechanisms behind cell-matrix physiochemical interactions and downstream cellular processes. Recently, Laura Brigo, Giovanna Brusatin and their co-workers reported a study on high-resolution 3D gelatin woodpile structures suitable for cell studies on the sub-micron scale. They found that, when particular fabrication conditions were selected (i.e., ~40 mW laser power on the sample plane and a scan speed of 150 mm/s), the final topological and mechanical properties of the polymerized gelatin structures were suitable for cell accommodation in the interior volume of the woodpiles. Interestingly, the human BJ cells Laura Brigo used in this research were capable of deforming the micron and sub-micron features of the high-resolution hydrogel of GelMA initiated by P2CK and crosslinked with PEGDA in terms of cell invasion within the rods and cell attachment between contiguous wood piles [37]. This feature showed the great potential of the TPP hydrogels for application in the studies of cell adhesion, deformation and migration on the microscale. 

### 4.2. Cell Differentiation

**Figure 10:**
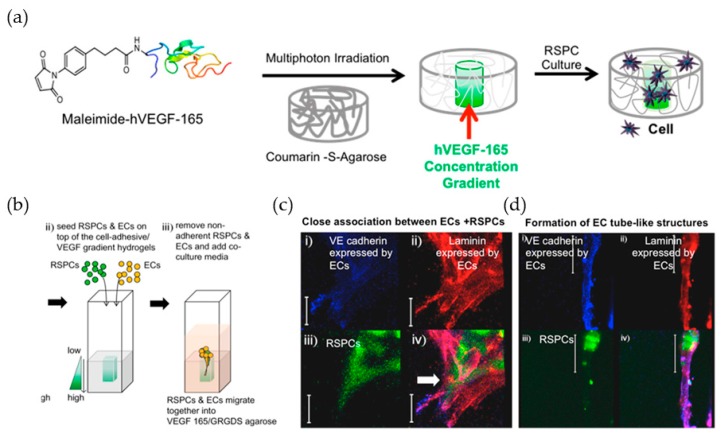
(**a**) Schematic representation of the maleimide-VEGF-165 concentration gradient in hydrogels; (**b**) Schematic of the co-culture system of ECs and RSCs in the VEGF-165 concentration gradient with uniform GRGDS immobilized; (**c**,**d**) Close interactions between the ECs and RSCs and the formation of EC tubular-like structures after 14 days in the presence of RSCs. Reproduced with permission from Reference [119].

Designing stimuli-responsive biomimetic hydrogels that can be used as extracellular matrices (ECMs) is essential for gaining insight into the mechanisms of cell-matrix and cell-cell interactions and stem cell differentiation. With photo cage or radical polymerization modification and multiphoton excitation systems, researchers have created more dynamic hydrogels with physiochemical properties that can be adjusted with external stimuli to better mimic the natural ECMs found in vivo. By controlling the spatial position of protein conjugation with on-demand two-photon excitation of photo labile groups, such as *o*-NB, Bhc and 3-(4,5-dimethoxy-2-nitrophenyl)-2-butyl ester (DMNPB), hydrogels can be fabricated with spatial and temporal resolution for cell differentiation research (Figure 10).

Molly S. Shoichet and her group focused on building biomimetic cellular microenvironments via two-photon patterning [55,56,118] of polysaccharide-based hydrogels modified by a Bhc motif [14] for exploring the effects of the ambient conditions on the interactions between retinal stem cells (RSC) and endothelial cells (EC) [119]. The researchers created Bhc-modified agarose hydrogels with a linear concentration gradient of photochemically immobilized VEGF165 and cell-adhesive peptide, glycine-arginine-glycine-aspartic acid-serine (GRGDS), to guide EC growth. From the advantages of two-photon patterning, this well-defined, spatially controlled co-culture platform demonstrated that ECs inhibited the proliferation and differentiation of RSPCs. The strategy of utilizing photo removable groups to immobilize a protein in a controlled gradient and the temporal activation of bioactive molecular provides new possibilities for the design of more complex biomimetic hydrogel systems to further the understanding of biological problems.

### 4.3. Vascularized Networks In Vivo

**Figure 11:**
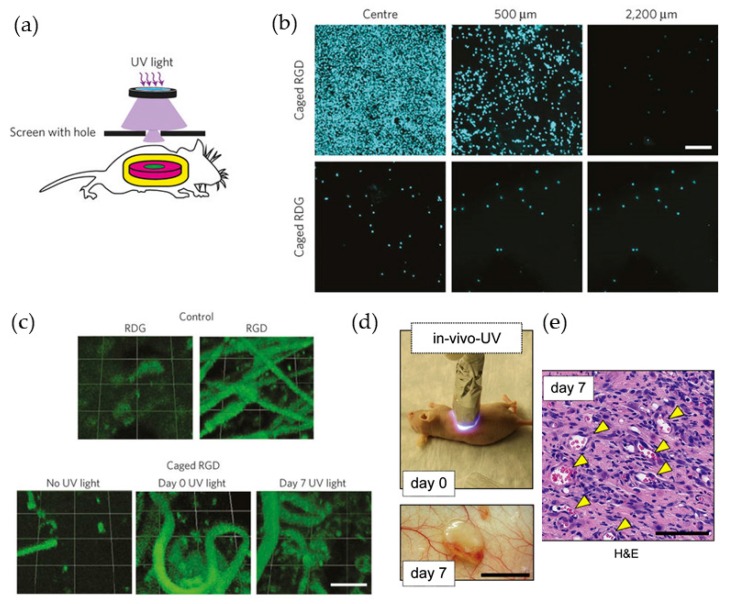
(**a**) Schematic representation of transdermal UV light exposure through a mask; (**b**) Photographs of explanted hydrogels stained for adherent cell nuclei for caged RGD and RDG presenting hydrogels at different distances from the center of irradiation (DAPI, color-coded cyan, scale bar, 40 µm); (**c**) PEG-maleimide hydrogels presenting peptides were implanted subcutaneously and exposed to UV light transdermally at selected time points (scale bar, 100 µm); (**d**) Representative images of a mouse receiving transdermal UV light at day 0 and the construct in the subcutaneous space after 7 days (scale bar 1 cm); (**e**) Representative H&E-stained section from a day 7 construct that was in vivo-UV polymerized (yellow arrowheads mark perfused blood vessels) (scale bars 50 mm). (**a**–**c**) reproduced with permission from Reference [13] and (**d**,**e**) reproduced with permission from Reference [14].

Photo polymerized hydrogel materials that are compatible with vascular morphogenesis form an active area of research in tissue engineering. Precise spatial and temporal control is essential for eliciting targeted cellular responses in those hydrogel materials in vivo. Currently, attention has been drawn to the strategy of spatiotemporally regulating the in vivo formation of biocompatible vascularized hydrogel networks with protecting groups via transdermal light exposure to uncover the activity of the implanted motifs or with the radicals generated from initiators within the mixture. Both those methodologies can provide a preferable means for delivering drugs, proteins and cells in applications requiring the formation of vascular networks in vivo (Figure 11). 

Juan M. Melero-Martin and his group injected liquid GelMA into immunodeficient mice and rapidly cured the liquid via transdermal exposure to UV light. After 7 days of cultivation in vivo, those bioengineered vascular networks formed functional anastomoses with the host vasculature. Moreover, varying the initial exposure time (15–45 s) showed a decrease in the vascular density and shrinkage of the average lumen size due to an increase in the crosslinking degree [14]. Andrés J. García, Ted T. Lee and their co-workers reported non-invasive transdermal light activation of the cell-adhesive peptides on implanted biomaterials to regulate the in vivo cell adhesion and spatial patterning, encapsulation and vascularization. The researchers modified the cyclic RGD peptide cyclo(Asp-d-Phe-Lys-Arg-Gly) with a 3-(4,5-dimethoxy-2-nitrophenyl)-2-butyl ester (DMNPB) photo labile caging group and conjugated it to PEGDA, which can be crosslinked under 365 nm UV light exposure via the presence of Irgacure 2959 (0.05%). Then, the researchers injected the hydrogel in liquid form into the skin of mice and trigged the biofunction with transdermal UV exposure. Several days later, robust blood vessel growth was observed via fluorescence observation. This UV light-labile cage strategy shows the capability of precise spatiotemporal bioligand incorporation and its widespread potential in biomedical applications [13].

## 5. Summary and Outlook 

We have briefly summarized the materials and chemistries applied to the synthesis of photo-based hydrogels. From radical polymerization to photo click chemistry, both direct and indirect light-curing strategies have been investigated. In addition, in contrast to the utilization of single- or multiphoton excitation of initiators or monomers, photo labile groups for photo degradation or photo cleavage are also discussed. Those methods have significantly promoted the number of biomimetic hydrogels in biomedically relevant research. In particular, hybrid processing, which combines the photo cleavage mechanism and rapid photo-free click reactions by caging the active groups with photo labile terminals or inserting photoactive sites, have greatly improved the probability of the involvement of light in the pre- and post-processing treatments and even the main fabrication procedure of biomimetic hydrogels. While, laser-based processing of hydrogels is often concomitant with limitations including swelling-related deformations and compromised spatial resolution. Those drawbacks refer to several aspects of the processing we reviewed before, including the swelling or transformation during the postproduction as well as the relatively low mechanical properties of the materials.

Considering this, the extrude-based 3D bioprinting, combing with fast photo crosslinking kinetic and predefined exposure program can fabrication complex hydrogel construct with various stiffness distribution and cell encapsulation capability [120]. Burdick and his group developed a generalizable bioprinting method to enable 3D printing of hydrogel structures form photo-crosslinkable precursors (MeHA and NorHA) [121]. They introduced the light though a silicone tubing to crosslink the hydrogel immediately prior to deposition and they termed as “in situ crosslinking”. With programed light intensity, exposure time and co-axils injector configuration, they fabricated various hydrogel construct, including core-shell, heterogeneous, or hollow tube. While, compared with the laser-based techniques, extruded bioprinting still hindered by its limited resolution in fabricating small volume, high complexity hydrogel construct [122]. 

Furthermore, it is notable that the interactions between photons and biomaterials in the design and functionality of hydrogels for biomedical purposes remain at a basic level. Scientists have investigated many significant effects of light on cell behavior, such as growth, proliferation and differentiation, from both positive and negative perspectives and these studies have shown the possibilities for engineering those features through more programmable and integral methodologies. 3D-printed microscale LED sources, modulable microfluidic biochemical platforms and biological circuits are powerful components for creating photosensitive micro devices with complex intrinsic structures, time-dependent actuation and self-healing or evolution capabilities based on an embedded biological core. Click chemistry with and without light as a catalyst has demonstrated great potential in the rational design of bioactive hydrogels. Besides, the development of photo initiator-free two-photo-induced coupling reactions could advance the field of biomedical hydrogels design and regenerative medicine. Recently, Carsten Werner observed an undescribed phenomenon in photochemistry that dictates reactivity of maleimide groups in two-photon mode [123]. Through their novel synthetic pathway, they realized the two-photon pathway in [2 + 2] cycloaddition of maleimides, which is a truly type of two-photo click chemistry. By combining the state-to-the-art engineering technologies, such as synthesis biological circuit [124], mechanical metamaterial design [125] and biomimetic 4D printing [126], the rapid reactions and bio-orthogonal characters of click chemistry will freshen the landscape of the design and functionality of biomedical hydrogels. 

## Figures and Tables

**Table 1:** Modifications of hydrogel backbones used for photo initiated polymerization.

Hydrogel Backbones	Acylated-Based Resources	Advantages	Drawbacks	Applications	References
Gelatin	Methacrylic anhydride, methacrylamide	Various modification degrees could be achieved by different concentrations of MA monomer solution Containing RGDs for cell adherence Mechanical tunability	Long-term outcomes deficiency Mechanical properties fading by degradation	Cell-encapsulating hydrogel Engineering Vascular Networks Tissue-Specific Differentiation	[21,24,34,35,36,37]
Hyaluronic acid	methacrylic anhydride, Glycidyl Methacrylate	Tunable volumetric swelling ratio, compressive modulus and degradation time Interacts with numerous cell surface markers, including CD44 and CD168	Low cell adhesion without extra modifications of sulfate derivatives Unexpected interaction with cell surface	Corneal laceration Cardiac repair Control of stem Cell Behavior	[26,27,38,39,40,41]
PEG	methacrylic anhydride	Hydrophilicity and relative inertness PEG can be modified with a wealth of different functional groups Commercial friendly	Need various modification for biofunction Average weight suit different situation specifically	Cell-encapsulation Cell mechanical behavior Vascular network strengthen	[23,42,43]

**Table 2:** Direct photo triggered reactions or post-gelation modification through thiol-ene click chemistry.

Mechanism	Nomenclature	Features	References
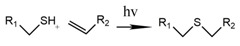	thiol-vinyl or thiol-(meth)acrylate	The presence of initiators increases the polymerization rates and acrylate conversion percentage.	[44,45]
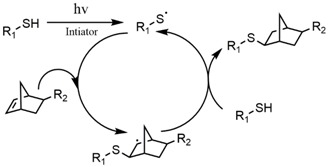	thiol-norbornene	Radical-mediated step-growth and biorthogonal polymerization	[32,46,47,48,49]

**Table 3:** General summery of hydrogel compatible light-induced fabricating techniques.

Techniques	Hydrogel Materials	Photon Chemistry	Advantages	Drawbacks	Resolution	References
Micropattern	PEGDA PEGDM GelMA HA MeTro	Crosslinking Degradation	Easy-to-use Easy equipment Straightforward	The entire thickness is with no control The inability to encapsulate cells Hard to find suitable exposure time	100 μm	[21,26,69,75,76,77]
(μ)SLA	GelMA HA HEMA	Crosslinking	High throughput Fully automated Substrate specific Complex 3D constructs High precision and resolution	Scaffold shrinkage due to water evaporation Incomplete conversion thus post-curing essential Limited availability of cells Non-homogeneous cell distributions Cytotoxic photo initiator Complex architectures with tunable micro- and macroscale features are difficult to achieve	5–30 μm	[78,79,80,81,82,83,84,85]
Two photon polymerizations (2PP)	Fibronectin Bovine Serum Albumin (BSA) PEG-DA	Crosslinking Degradation	Have a focal spot Higher resolution	Time consuming adjustment to new materials Not able to fabricate large scaffolds High capital and operating costs Operating systems are relatively slow	0.5–1 μm	[85,86,87,88]

**Table 4:** Advances on the improvement of TPP.

Modified Aspect	Hydrogel Applied	Operations	Results	Technology Improvement	References
Material	(Gel-MOD)/(Gel-MA)	Modify the primary amines as well as the carboxylic acids from gelatin with photo-cross-linkable moieties	Superior and tunable mechanical properties	Strengthen the stiffness of single cured voxel, leading to a higher precision and efficiency	[115]
Material	HA	HA is covalently cross-linked with poly (ethylene glycol) diacrylate (PEGDA) in situ	Modulate the physical and chemical properties	Generate porous scaffold More efficiency	[78,116]
Technology	Resin	Involve holographic data with a spatial light modulator (SLM)	Multiple foci 2PP technique	Higher precision; Shorter fabrication times	[117]
Material	Chitosan	*n*-succinylated followed by a conjugation with glycidyl methacrylate	Convert in a water-soluble species	Higher precision; Generate a diversity of tailor-made scaffold shapes	[114]

## Supplementary Materials

Supplementary File 1
