# Supplementary material for: Photo Processing for Biomedical Hydrogels Design and Functionality: A Review
Source: Polymers (Basel). 2017 Dec 22;10(1):11. doi: 10.3390/polym10010011 (PMC6415176; doi:10.3390/polym10010011)
Supplement: Supplementary File 1 [file polymers-10-00011-s001.pdf]

DATA SHEET

PHOTONIC PROFESSIONAL *GT*

The Photonic Professional *GT* is the world's highest resolution 3D printer. Based on two-photon polymerization (2PP), it allows for additive manufacturing and maskless lithography with the same device. Submicrometer resolution printing with feature sizes down to 200 nm and optical quality surface finishes are characteristic key features. Two powerful writing modes move the laser focus with respect to the photoresist: A piezo-mode for arbitrary 3D trajectories and a galvo-mode for ultra-fast structuring in a layer-by-layer process. With these unique features, the versatile system covers the broad demands encountered in nano-, micro- and mesoscale fabrication.

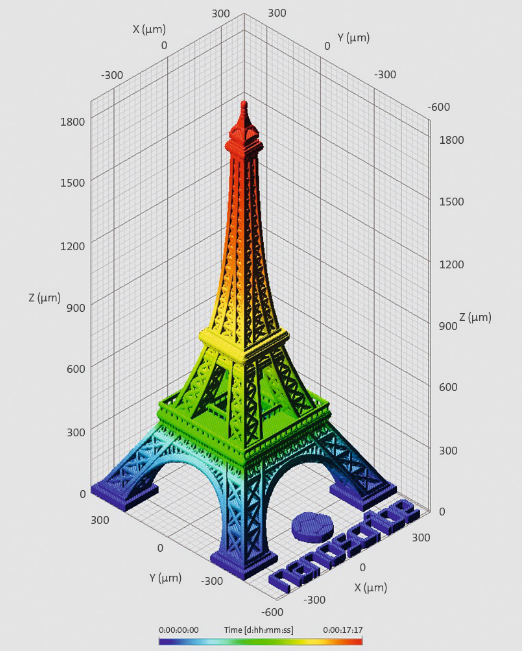

Screenshot of print job editor DeScribe

SYSTEM & COMPONENTS

The details of the turnkey system, its comprising components and available options are listed below and highlighted in the image.

OPTIONS, ACCESSORIES & CONSUMABLES

The following options, accessories and consumables are available to tailor the performance of the systems to application- and environment-specific requirements:

- Automatically exchangeable scanning objectives*
- Diverse substrate holders and substrates*
- Standard & cleanroom configuration*
- 2PP-optimized photopolymer resins from our IP product lineup

STARTER KIT

All systems are delivered with a starter kit consisting of a supporting rack for the convenient placement of sample holders, beakers with a substrate holder for wet-chemical development, and a selection of Nanoscribe's IP photopolymer resins.

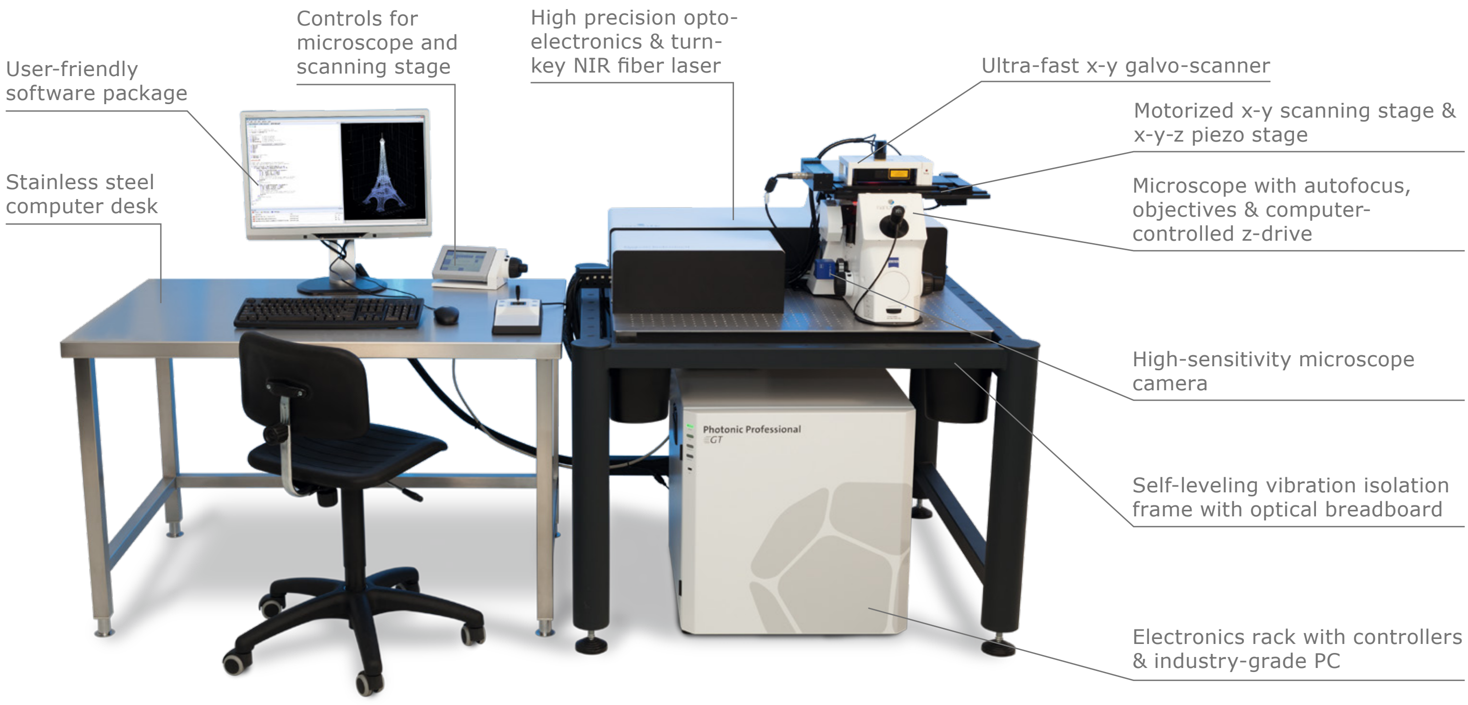

* *more data available on request*

**Technical details**	**Photonic Professional *GT***
**Printing performance***	
minimum 3D lateral feature size	200 nm (spec.); 160 nm (typ.)
finest 2D lateral resolution	500 nm (spec.); 400 nm (typ.)
finest vertical resolution	1,500 nm (spec.); 1,000 nm (typ.)
beam scanning speed**	10 mm/s (typ.)
piezo scanning speed**	100 µm/s (typ.)
accessible print area by motorized stage	100 x 100 mm²
x-y-z piezo range	300 x 300 x 300 µm³
x-y galvo scan range	200 - 600 µm Ø dependent on scanning objective***
maximum object height fine	300 µm
maximum object height coarse	3 mm
**Laser source & safety**	
laser source	NIR femtosecond laser
laser safety	class 1 as complete system according to EN 60825-1:2007
**Software package**	
NanoWrite	graphical user interface controlling the system
DeScribe	print job editor including STL/DXF import and process preview
**Electrical properties**	
rated voltage range	AC 100 - 240 V
rated frequencies	50 / 60 Hz
rated current	5 A (max.)
average power consumption	< 500 W
main supply overvoltage	category II
grounding equipment conductor	required
electrical safety	in accordance with EN 61010-1:2010
**Weights and measures (w/o working desk)**	
total weight	320 kg
dimensions (W x L x H)	90 x 90 x 142 cm³
weight (system w/o electronics rack)	225 kg
dimensions (electronics rack) (W x L x H)	56 x 60 x 64 cm³
weight (electronics rack)	95 kg
minimum wall distance	30 cm
**Ambient conditions**	
altitude	2,000 m (max.)
operating temperature	22° C (± 4° C)
temperature stability	± 1° C
storage temperature	20° C (± 10° C)
relative humidity	60 % (max.)
air pressure supply for vibration isolation table	4 - 6 bar
room lighting	yellow light required****

* *Optimum achievable performance depends not only on the machine and its configuration but also on the photoresist used. Please contact us for more specific information regarding your process.*

** *Effective printing time depends on more aspects than merely the piezo's or the laser beam's scanning speed; smart software and control features as well as process tailored materials make the Photonic Professional GT the fastest and most precise 3D printer on the nano-, micro-, and meso-scale; please consult us for more details.*

*** *More data available on request; please consult us for the proper choice of scanning objectives that match your application.*

**** *Light tubes (e.g. OSRAM L 36 W/62 Yellow) or UV-filter tubes (e.g. ASR-LY5-UV (Metholight)) or any alternative yellow light sources*

CONTACT

Nanoscribe GmbH
Hermann-von-Helmholtz-Platz 1
76344 Eggenstein-Leopoldshafen
Germany

Phone +49 721 981 980 0
Fax +49 721 981 980 130
E-Mail sales@nanoscribe.com

www.nanoscribe.com
www.facebook.com/nanoscribe
www.youtube.com/nanoscribe

DS/GT/V04_2016
